# Cool Air Etc., in Measles and Scarlet Fever

**Published:** 1882-09

**Authors:** Thos. N. Reynolds


### Cool Air, Etc., in Measles and Scarlet Fever.

Many children die or recuperate badly in measles and scarlet fever from want of fresh air. The same obtains in other affections, also, but proportionately perhaps oftener in these two of the major exanthemata.

The people imagine heat indispensable, and they dread draughts. Physicians advise a warm atmosphere, sometimes to favor eruption on the surface, to protect the air passages in measles and the kidneys in scarlet fever, and the result is often bad air for respiration. Equable heat to the body is necessary, but should be maintained by the clothing, or artificially, in the bed, and not by raising too high the temperature of the room. Better have it rather low than too high, provided the body be kept warm.

Hot air is not generally necessary for the bronchial affection in measles. I have seen a case do well in a room that froze water at night, and have come to think lately that a somewhat cool room is best always in measles and scarlet fever, whether sthenic or asthenic. Not over 60° Fahrenheit in winter is better, and as cool as possible in summer, with very free ventilation.

In scarlet fever especially, cool fresh air is essential to promote active interchange of the normal gases in the lungs, and elimination through them, also, of some of the poison. It relieves restlessness, and is sedative, because grateful, to the patient. It is anti-pyretic somewhat from mere presence in the nares and throat; and nothing is so decided a vaso-motor stimulus as cool, fresh air when supplied for respiration in place of the hot and impure. It gives firmness and efficiency to the whole circulatory apparatus.

Efficient capillary and venous circulation favors secretion and excretion, and relieves internal congestions, in scarlet fever necessary in the glomerular and intertubular capillaries of the kidneys, and the automatic nervous ganglia of the body and brain.

In both these affections the stomach and bowels should have attention at first; patients are usually taken in full habit, the temperature rises, the secretions are checked, and the bowels should be moved at once. Sometimes they have moved sufficiently, but generally not. Solid food should then be withheld till the eruption is over. Little more than small drinks of water should be given in sthenic cases for the first two or three days.

Medicines are rarely necessary in ordinary uncomplicated measles, except to please friends.

In scarlet fever, where the temperature is greater, I give vegetable acids, diluted with water, freely for the first few days, or whenever there is thirst, and as long as they agree with the stomach and bowels. Citric acid is good in the form of lemonade, without sugar. I prescribe for a child four or five years old:

℞ Acidi acetici diluti.............................. ℥ ss.
Aquæ ad.............................................. ℥ iv.
Misce et fiat Mistura.

Sig. Teaspoonful in water every hour, if awake.

It proves, in a degree, diuretic, diaphoretic, antipyretic, and sedative, and promotes elimination from the alimentary mucous surface. It increases destructive metamorphosis, especially adipose, so desirable in the fleshy. This, with its stimulus to elimination, tends to lower heat of body; and renders it, to a certain extent, curative, as well as an agreeable drink. It is a sedative largely from allaying the thirst.

Mineral acids do none of those things so well, and are not so appropriate during the stage of eruption.

Digitalis is beneficial in venous distension and weak cardiac contraction.

Quinine is essential in malarial complication, and generally useful in high temperature and extreme septicæmia, but need not be resorted to at first, and rarely at all in scarlet fever.

The cold bath is not generally without some risk from shock or imperfect reaction; but extreme temperature may be lowered somewhat by cold to the head and palms of the hands, and occasional quick sponging with cold water, followed by friction.

We close with the end of the eruptive stage; for the acute tubal nephritis, median otitis and lymphadenitis, if they happen to follow, form new considerations, and are the principal results, besides death, we try to prevent.—*Thos. N. Reynolds, M. D., in Trans. Mich. Med. Soc. and So. Prac.*

